# Dry Eye Disease in Routine Rheumatology Practice

**DOI:** 10.31138/mjr.29.3.127

**Published:** 2018-09-27

**Authors:** Sevastiani Ziaragkali, Aggeliki Kotsalidou, Nikolaos Trakos

**Affiliations:** 1 Department of Rheumatology, Hygeia Hospital, Athens, Greece,; 2 Op Eyelids, Lacrimal & Orbital Department, Hygeia Hospital, Athens, Greece

**Keywords:** Dry Eye Disease, Ocular Surface, Sjögren’s Syndrome, Rheumatic Disease

## Abstract

Dry eye disease (DED) is one of the most frequent ophthalmological conditions, with a major impact on patients’ quality of life. Tear film instability and tear hyperosmolarity are considered to play a crucial role in the vicious cycle of dry eye disease. They occur as a result of, either a reduced lacrimal secretion or an excessive evaporation from the tear film. There is a well-known association of DED, not only with autoimmune diseases but also with other systemic diseases and medication. Early diagnosis is important and it is based on the presence of classical symptoms and signs of dry eye in combination with specialized methods. The comprehension of the pathophysiology is significant, as different approaches can be taken to treat DED, depending on the cause and primary source of the disease, as well as on disease severity.

## INTRODUCTION

DED is a significant global health problem and one of the most common ophthalmological conditions seen in clinical practice. It consists of qualitative and quantitative alteration of the tear film, whose main role is lubrication, nutrition, optical transparency, cleanliness and a defense mechanism of the ocular surface.^1^ Tear film instability, which is recognized as the “hallmark” of the vicious cycle of DED, leads to inflammation and apoptosis on the ocular surface. This can occur as a result of a heterogeneous group of conditions with multifactorial etiologies and there is a well-known association of DED, not only with rheumatic diseases like Sjögren’s Syndrome (SS), Rheumatoid Arthritis (RA), Systemic Sclerosis (SSc) and Systemic Lupus Erythematosus (SLE), but also with several systemic diseases, including lymphoma, amyloidosis, hemochromatosis and sarcoidosis.^2^

DED is a significant public health issue. More than 20 million Americans have DED.^1^ However, the exact prevalence of dry eye has proven difficult to obtain, mainly because of the failure for standardized definition and diagnostic criteria used to define the disease and the lack of a consensus on which tests should be used to confirm the diagnosis.^3^ An additional difficulty is the lack of correlation between patients’ irritative symptoms and the result of the tests.^4^ The prevalence of DED, with and without symptoms, ranged in prevalence from 5% to 50%. DED prevalence based on signs alone was generally higher and more variable, reaching up to 75% in a part of the population. Criteria for positive DED signs varies between studies and it is acknowledged that some signs may reflect secondary outcomes or may be related to normal aging. It is confirmed that symptomatic disease and signs of DED increase with age and higher rates of DED are reported in women than men, although the differences generally become significant only with increasing age.^3^

DED results in significant costs that fall on individuals, health service and the wider economy. The economic burden is significant. Estimated global sales of artificial tears exceeded US $540 million annually in 2002, while Galor et al. estimated that the mean prescription medication expenditure per patient per year was 299 $ in USA in 2005.^5^ DED has a devastating impact on patient’s overall quality of life. Patients with dry eye experience symptoms of ocular discomfort, dryness and episodic visual disturbances. The disease in not only uncomfortable but interferes with ability to work and carry out daily functions. It is associated with lower professional work performance, decreased quality of life and poor general health. Several studies have indicated that the health-related quality of life (HRQoL) burden increases with the severity of disease and these patients had HRQoL scores in the range of conditions such as III/IV angina and renal dialysis.^6^ Dry eye patients with rheumatic disease were more likely to suffer from depression and anxiety.^7^

The known risk factors for DED are summarized in **Table 1**.^3^

**Table 1.** Risk factors for DED

**Consistent**	**Probable**	**Inconclusive**

Age Gender Race Meibomian Gland Dysfunction Connective Tissue Disease Sjögren Syndrome Androgen Deficiency Computer Use Contact Lens Wear Estrogen Replacement Therapy Hematopoietic Stem Cell Transplantation Certain Environmental Conditions (Pollution, low humidity, and sick building syndrome) Medication use (Antihistamines, antidepressants, anxiolytics, and isotretinoin)	Diabetes Rosacea Viral Infection Thyroid Disease Psychiatric Conditions Pterygium Low Fatty Acid Intake Refractive Surgery Allergic Conjunctivitis Additional Medication (Anti-cholinergic, Diuretics, b-blockers)	Hispanic Ethnicity Menopause Acne Sarcoidosis Smoking Alcohol Pregnancy Botulinum toxin injection Multivitamins Oral contraceptives

### The Ocular Surface / Anatomy – Physiology

The ocular surface and tear-secreting glands are a complex integrated functional system that are interconnected by sensory and autonomic nerves.^8^ The ocular surface is covered by a continuous sheet of epithelium, lining the cornea, the anterior globe and tarsi, and extending to the mucocutaneous junctions of the lid margins. Hydration of the ocular surface is maintained by the tears, which bathe it continuously and provide an unbroken film over its exposed surface. The tears are secreted mainly by the lacrimal glands, with additional contributions from the conjunctiva, including the goblet cells and Meibomian glands of the eyelids.

The total lacrimal tissue mass consists of the main lacrimal gland, which is a tubuloacinar, serous gland composed primarily of acinar, ductal and myoepithelial cells, with the acinar cells comprising 80% of the total, and the accessory glands, which constitute about 10% of the total lacrimal tissue. The accessory glands consist of the accessory glands of Krausse in the upper and lower fornix and the accessory glands of Wolfring in the upper and lower eyelids. The lacrimal gland is richly supplied by immune cells that occupy the interstitial space. Plasma cells (Ig)A+ predominate, T-cells are the next most common cells and B-cells are found in smaller number.^9^ The lacrimal glands produce the aqueous layer, which is the main layer of the tear film and constitutes the thickest portion of the tear film, representing almost 90% of its total volume. It is the layer most frequently affected by pathologies that lead to DED.^10^

The conjunctiva is a mucous membrane with a lamina propria (stroma) of loose connective tissue covered by an epithelium that is kept permanently moist. It is recognized not only as a barrier against the outer environment but also for its secretory and immunoprotective function. The conjunctival epithelium consists of two cell types – epithelial cells and goblet cells, both deriving from the same conjunctival stem cell. The conjunctival epithelium cells produce water, electrolytes, mucins and functional proteins such as lubricin. They also produce integral membrane mucins that constitute the superficial glycocalyx of the cell which is necessary for the wettability of the epithelium and acts as a lubricant that reduces friction at the ocular surface and as an anti–adhesive that combats microbial colonization. Additionally, the conjunctiva epithelial cells contain transmembrane water channels (aquaporins) concerned with water movement between the conjunctiva and the aqueous phase of the tear film, which is a substantial part of the mucus layer, the tear film layer intimately related to the surface of the eye.

Conjunctival goblet cells play a significant role in tear film stability. They package and secrete the gel – forming mucin, MUC5AC. Gel mucins have an enormous water–binding capacity and transform the aqueous tear into a mucoaqueous gel that makes up the main volume at the tear film and offers hydration at the ocular surface. Mucins also have a lubricative function at the lid – globe interface that is important for movements of the eyeball relative to the eyelids. Furthermore, the mucin of the mucoaqueous layer has protective properties: binding microorganisms and inhibiting their attachment to the epithelium, and also binding sIgA, several antimicrobial proteins and peptides.

The meibomian glands are modified sebaceous, holocrine glands, and richly innervated with sensory, sympathetic and parasympathetic nerves. Their secretory product (meibomian lipid) is delivered into a shallow reservoir on the skin of the eyelid margin, just anterior to the mucocutaneous junction, and is spread onto the preocular tear film with each blink.^9^ The Meibomian lipid composes the superficial lipid layer of tear film. The part of the lipid layer adjacent to the aqueous-mucin gel is composed of hydrophilic polar lipids, including the amphipathic lipid O-acyl-w-hydroxy-fatty-acid, phospholipids, ceramides and cerebrosides, whereas the bulk of the tear lipid layer consists of overlying non-polar hydrophobic lipids, including wax esters, cholesterol, esters, triglycerides, free fatty acids anal hydrocarbons that are associated with the polar phase by means of hydrophobic bonds.^10^ The tear film lipid layer plays a significant role in stabilizing the tear film and has been considered until recently to provide a barrier to tear evaporation. However, some precious and more recent studies have suggested that it reduces evaporation by no more than 10%.

Tear film, besides water and electrolytes, also contains proteins including growth factors such as epidermal growth factor (EGF) and hepatocyte growth factor (HGF), that are essentials for the maintenance of the epithelium, and defense proteins such as lysozyme, lactoferrin, surfactant protein-D and trefoil peptide, concerned with innate immunity, and sIgA. These proteins, such lysozyme and lactoferrin, are decreased in DED, making the eye more vulnerable to infection.^9^

Any disturbance to any of the above-mentioned components of the ocular surface and subsequently to tear film layers results in the development of DED with the consequent cascade of proinflammatory factors that cause the known symptoms of the condition.

## Lacrimal functional unit (LFU)

The ocular surface (cornea, conjunctiva and eyelids as well as main and accessory lacrimal grands and Meibomian glands) and the interconnecting neural reflex arcs function as an integrated unit, the LFU, with communication between these compartments occurring through their secondary / autonomic neural reflex loop.^11^

The afferent limb of the reflex arc arises in the trigeminal innervation of the ocular surface epithelia, including cornea, conjunctiva and eyelids, whose central endings synapse with neurons in the superior salivatory nucleus in the brain system, probably adjacent with the 7^th^ cranial nerve.

The efferent limb of the reflex arc is a parasympathetic pathway, whose secretomotor, preganglionic fibers arises in the superior salivatory nucleus. These fibers exit the pons by the nerves intermedius of the 7^th^ cranial nerve and reach the pterygopalatine ganglion via the pterygoid nerve. Then, the postganglionic fibers reach the lacrimal gland via the lacrimal nerve. The nasolacrimal passage is also considered to contribute to this reflex system. Another reflex arc that contributes to protect the ocular surface is that subserving the blinking. Blinking plays a key role in tear dynamics by spreading, mixing and distributing the tears and clearing cellular and other debris. Consequently, the blink interval is recognized as chief modifier of evaporation, likely determining the set point of tear osmolarity.^9^

So, tear film stability and tear osmolarity, hallmarks of the normal eye, are determined by these reflexive mechanisms, and are threatened when the interactions in LFU are compromised by decreased tear secretion, delayed clearance and altered tear composition.

### The Vicious Circle of DED

The core mechanism of DED is tear hyperosmolarity, which is the hallmark of the disease. It damages the ocular surface both directly and by initiating inflammation. Tear hyperosmolarity stimulates a cascade of events in the epithelial cells of the ocular surface, involving MAP kinases and NFκβ signaling pathways and the generation of inflammatory cytokines IL-1 and TNF-a, and proteases such as MMP9. Experimentally, the expression of IL-1, IL-6 and TNF-a by ocular surface epithelia is critical to the inflammatory response of DED.

A step in the amplification process is the generation of signals that recruit both innate and adaptive inflammatory cells to the site of inflammation. Another critical step in the homing of these inflammatory cells to the ocular surface is the expression of endothelial adhesion molecules, such as the intracellular adhesion molecule-1 (ICAM-1), which is expressed by conjunctival and corneal epithelium. ICAM-1 is an adhesion molecule that binds to inflammatory cells expressing the ligand, LFA-1 (integrin leukocyte functional antigen 1), causing rolling, transmigration and activation at the site of inflammation. ICAM-1 represents a therapeutic target for the treatment of DED as Lifitegrast, an ICAM inhibitor has been approved by the FDA. All these inflammatory molecules activate and recruit inflammatory cells to the ocular surface, which become an additional source of inflammatory mediators.

Three distinct cell types are involved in the innate inflammation response: neutrophils, NK (natural killer) cells and monocytes/macrophages. A new mechanism leading to tissue damage in DED has been identified, involving the release of DNA into the tears from desquamating ocular surface epithelial cells and invading neutrophils. This extracellular DNA can, on its own, or combined with components of neutrophil origin, cause direct damage to the ocular surface. As with regard to NK-cells, they may be an early source of interferon-γ (IFN-γ) that is responsible for the activation and the differentiations of T-helper 1(Th1) T-cells, inductions of costimulatory signals by antigen-presenting cells (APCs) and which itself is a key inflammatory cytokine causing conjunctival epithelial damage, goblet cell loss and damage to the epithelial glycocalyx. Goblet cell loss is a feature of every form of DED, reflected by reduced tear levels of MUC5AC. Furthermore, the contribution of adaptive immunity initiated by antigen presentation in ocular surface inflammation is recognized. Although the antigens that initiate this process in DED are not known, the expression of auto-antigens is hypothesized to be a key trigger to the inflammatory epitheliopathy in SS.^9^

Additionally, it is hypothesized that DED is a metabolic disorder characterized by an imbalance of Ω-3 and Ω-6 polyunsaturated fatty acids leading to the underproduction of preresolving lipid mediators, as Ω-3 group are precursors of eicosanoid with potential anti-inflammatory effects and Ω-6 group has proinflammatory effects.^12^

The result of all these mechanisms is the characteristic punctate epitheliopathy of DED and a tear film instability which leads at some point to early tear film breakup. This breakup exacerbates and amplifies tear hyperosmolarity and completes the vicious circle of DED.

### Definition and Classification of DED

TFOS DEW II has redefined dry eye as follows:

“Dry eye is a multifunctional disease of the ocular surface characterized by a loss of homeostasis of the tear film, and accompanied by ocular symptoms, in which tear film instability and hyperosmolarity, ocular surface inflammation and damage, and neurosensory abnormalities play etiological roles”.

The current definition has concluded, besides tear film instability, hyperosmolarity and inflammation, neurosensory abnormalities, which have featured increasingly in the recent literature.

The current classification indicates two major subtypes of DED: Aqueous Deficient Dry Eye (ADDE) and Evaporative Dry Eye (EDE). Although it is recognized that these subtypes of DED may coexist, once tear break up occurs within the blink interval, an additional EDE component is added to the dry eye regardless of the initiative cause.^9^

#### Aqueous Deficient Dry Eye (ADDE):

ADDE implies that the tear hyperosmolarity results from a reduced lacrimal secretion in the presence of a normal rate of tear evaporation. ADDE is subdivided into Sjögren’s Syndrome DE (SSDE) and non-Sjögren’s Syndrome DE (NSDE).

Sjögren’s Syndrome is a chronic autoimmune disorder characterized by immune cell infiltration of exocrine glands (exocrinopathy or epithelitis) and systemic complications due to autoantibody production, immune complex deposition and lymphocytic infiltration of many organs.^13^ There are two forms of SS. In primary SS, ADDE syndrome occurs in combination with symptoms of dry mouth in the presence of autoantibodies, with evidence of reduced salivary secretion and with a positive focus score on minor salivary gland biopsy. In secondary SS, the features of primary SS occur together with the features of an overt autoimmune connective tissue disease; most commonly RA, SLE, systemic sclerosis, Primary biliary cholangitis (PBC) or mixed connective tissue disease.^10^ Although more recently, the American College of Rheumatology has recommended that the diagnosis of SS should be given to any patient who fulfills the diagnostic criteria of SS without distinguishing it as primary or secondary, recognizing them both to be a manifestation of immune dysregulation. However, the older terminology is still widely used.^9^

NSDE include acquired and congenital forms of DED where the systemic autoimmune features of SS have been excluded. The most common form of NSDE is age-related ADDE and corresponds to the term keratoconjunctivitis sicca. The clinical features resemble those of SSDE, but in general, age of onset is later, the degree of lacrimal gland infiltration lower, the rate of progression lower and severe disease less common than SSDE. Aging is the key in the pathology of age-related DED.^9^ According to Rocha et al., theories of aging may be classified as Programmed (involving genetic, hormonal and immunological influences) and Damage or Error-based, involving wear and tear, tissue oxidation and cross-linking, post-translational modification, or the consequence of somatic mutation.^14^ The potential contribution of tissue aging to this disorder could be due to a failure of any of the elements of the LFU, such as a loss of sensory drive from the ocular surface, reduced delivery of secretory neurotransmitters and loss of functional secretory tissue. At last, it seems that oxidative stress plays an important role in age – related NSDE.

Other causes include:

1. *Intrinsic lacrimal deficiency*: lacrimal ablation, congenital alacrima and triple – A syndrome (Congenital Alacrima, Achalasia, Addisson’s disease and autonomic dysfunction).^9^
2. *Inflammatory and other infiltrative conditions of the lacrimal gland*: Lacrimal secretion may fail because of inflammatory infiltration of the gland in sarcoidosis or lymphoma.^10^ Specifically, sarcoidosis is a chronic systemic disorder with an estimated prevalence ranging from 1 to 40 cases per 100.000 and it is characterized by the presence of non- caseating glanulomas in multiple organs. Lacrimal and salivary involvement is frequent, and it is typical for these patients to show significant enlargement of the gland. Regarding viral infections, sicca symptoms are present in 10% of patients with hepatitis C and up to 38% of patients who are HIV positive.
3. *Lacrimal gland obstruction*: Obstruction of the main palpebral and accessory lacrimal glands leads to ADDE and may be caused by any form of cicatrizing conjunctivitis and extensive conjunctival scarring such as chronic graft – versus – host disease (GVHD), Stevens-Johnson Syndrome/Toxic Epidermic Necrosis, mucous membrane pemphigoid and trachoma, and also after physical and chemical injury. Ocular GVHD is also worth mentioning. DED is a major late complication and occurs in 40–80% of patients after allogeneic hematopoietic stem cell transplantation. The ocular features of GVHD are complex and involve an interaction between the lacrimal and meibomian glands and the ocular surface. A key in its pathophysiological mechanism may be epithelial-mesenchymal transition (EMT), a process whereby epithelial cells are converted into multipotent mesenchymal stem cells that can differentiate into a variety of cell types. In GVHD-related DED, cross-reactions between the donor and recipient immunes cells generates a “cytokine storm”, which compromises the mucosal banners on the ocular surface and may trigger EMT at various sites. In the lacrimal gland, under the influence of local T-cells, EMT affecting myoepithelial cells is considered to cause severe fibrosis, resulting in gland loss and lacrimal duct obstruction.^9^
4. *Reflex hyposecretion*: A reduction in corneal sensitivity occurs in contact lens wearers, following LASIK refractive eye surgery and in diabetes mellitus, and reduces the sensory drive from the exposed ocular surface, reducing both reflex lacrimal secretion and the blink rate (increasing evaporative loss). Central damage to the facial nerve, involving the nervus intermedius, also leads to DED.^15^
5. *The role of medication* in causing or aggravating DED is complex and controversial. So systemic medication as topical ocular medication may induce DE. Especially for topical medication, long-term use of topical medications with preservative Benzalkonium Chloride (BAK) is important. Furthermore, oral polypharmacy needs to be studied more as it seems to be a cause of DED. Systemic and topical ocular medications that probably cause DED are included in **Tables 2** and **3**.^16^

**Table 2.** Systemic drugs that may cause or aggravate dry eye.

Class	Examples

Antihypertensive agents (beta-agonists)	Acebutolol
Antihypertensive agent (alpha-agonists)	Atenolol
Antiarrhythmic agents (beta blockers)	Carvedilol
	Labetalol
	Metoprolol
	Nadolol
	Pindolol
	Clonidine
	Prazosin
	Oxprenolol
	Propranolol

Antipsychotic agents	Chlorpromazine
	Fluphenazine
	Lithium carbonate
	Perphenazine
	Prochlorperazine
	Promethazine
	Quetiapine
	Thiethylperazine
	Thioridazine
	Brompheniramine
	Carbinoxamine
	Chlorphenamine (chlorpheniramine)
	Clemastine
	Cyproheptadine
	Dexchlorpheniramine

Bronchodilators	Diphenhydramine
Antispasmodics/Antimuscarinic	Doxylamine
Antiarrythmic agents	Ipratropium
	Atropine
	Homatropine
	Tolterodine
	Hyoscine (scopolamine)
	Hyoscine methobromide (methscopolamine)
	Disopyramide

Antineoplastic agents	Busulfan
	Cyclophosphamide Interferon (alpha, beta, gamma, or PEG)
	Vinblastine
	Cetuximab
	Erlotinib
	Gefitinib

Antihistamines	Cetirizine
	Desloratadine
	Fexofenadine
	Loratadine
	Olopatadine
	Tripelennamine

Antidepressants	Citalopram
	Fluoxetine
	Fluvoxamine
	Paroxetine
	Sertraline

Antileprosy agents	Clofazimine

Antirheumatic agents/analgesics	Aspirin
	Ibuprofen

Sedatives and hypnotics	Primidone

Drugs secreted in tears	Aspirin
	Chloroquine
	Clofazimine
	Docetaxel
	Ethyl alcohol
	Hydroxychloroquine
	Ibuprofen
	Isotretinoin

Antiandrogens	Tamsulosin
	Terazosin
	Doxazosin
	Alfuzosin

Neurotoxins	Botulinum A or B toxin

Antimalarial agents	Chloroquine
	Hydroxychloroquine

Retinoids	Isotretinoin

**Table 3.** Topical ocular drugs that may cause or aggravate dry eye.

Class	Examples

Agents used to treat	Betaxolol
Glaucoma	Carteolol
	Levobunolol
Beta-block	Metipranolol
	Timolol
Adrenergic agonist drugsing agents	Apraclonidine
	Brimonidine
Carbonic anhydrase Inhibitors	Brinzolamide
	Dorzolamide
Cholinergic agents	Pilocarpine
Prostaglandins	Bimatoprost
	Latanoprost
	Travoprost
	Dipivefrine
	Unoprostone
	Ecothiopate

Agents used to treat allergies	Emedastine
	Olopatadine

Antiviral agents	Aciclovir
	Idoxuridine
	Trifluridine

Decongestants	Naphazoline
	Tetryzoline

Miotics	Dapiprazole

Mydriatics and cycloplegics	Cyclopentolate
	Tropicamide
	Hydroxyamfetamine

Preservatives	Benzalkonium chloride

Topical local anesthetics	Cocaine
	Proxymetacaine
	Tetracaine

Topical ocular NSAIDs	Bromfenac
	Diclofenac
	Ketorolac
	Nepafenac

#### Evaporative Dry Eye (EDE):

EDE implies that tear hyperosmolarity is the result of an excessive evaporation from the tear film in the presence of normal lacrimal function.^10^ However, it is recognized that all forms of DED are evaporative in the sense that tear and ocular surface hyperosmolarity can only arise in response to evaporation. According to the TFOS DEWS report, EDE comes about as a result of a loss of evaporative banner function of the tears or due to reduced ocular surface wettability. This has led to a subclassification into eyelid-related EDE and ocular surface-related. Some of the causes of EDE are increased interpalpebral aperture such as in exophthalmos due to Thyroid Eye Disease, Lagophthalmos due to Facial Nerve Palsy or incomplete closure during bedtime such as nocturnal lagopthalmos, ectropion, lower eyelid laxity, decreased blinking rate such as in PC users, etc. All causes of DED have been included in **Table 4**.^9^

**Table 4.** Causes of DED.

**AQUEOUS-DEFICIENT DRY EYE (ADDE)**
Sjögren Syndrome Dry Eye (SSDE)
- associated systemic diseases
Rheumatoid arthritis
Polyarteritis nodosa
Systemic lupus erythematosis
Wegener granulomatosis
Systemic sclerosis
Primary biliary cirrhosis
Mixed connective tissue disease
Non-Sjögren Syndrome Dry Eye (NSDE)
Intrinsic Lacrimal Gland Deficiency
Lacrimal gland ablation
Congenital alacrima
Triple A syndrome
Age-related ADDE dry eye
Inflammatory and Other Lacrimal
Gland Infiltration
Sarcoidosis
Lymphoma
Viral Infection
Radiation Injury
Lacrimal Gland Obstruction
Cicatricial Conjunctivitis
GVHD
Stevens-Johnson Syndrome/TEN
Mucous Membrane Pemphigoid
Cicatricial pemphigoid
Pemphigus
Trachoma
Chemical injury
Hyposecretory States-Failure of the Lacrimal Functional Unit
Reflex Afferent Block
Topical anesthesia
Trigeminal nerve injury
Refractive surgery
Neurotrophic keratitis
Secretomotor Block
Parasympathetic damage
Pharmacological inhibition
Combined Afferent and Efferent
Block
Familial dysautonomia
Other Disorders
Meige Syndrome
Diabetes Mellitus
Pseudoexfoliation
**EVAPORATIVE DRY EYE**
Meibomian Gland Diseases
Lid-Related
Meibomian Gland
Dysfunction (MGD)
Primary
Meibomian seborrhea
Obstructive MGD
Cicatricial/non-cicatricial
Secondary to Local
Disease
Anterior blepharitis
Ocular surface
inflammation
Contact lens wear
Secondary to Systemic
Dermatoses
Rosacea
Seborrheic dermatitis
Atopic dermatitis
Ichthyosis
Psoriasis
Secondary to Chemical
Exposure
13-cis retinoic acid
Polychlorinated
biphenols
Antiandrogens
Genetically Determined
Meibomian Gland Diseases
Meibomian Agenesis and Dystichiasis
Anhydrotic Ectodermal Dysplasia
Ectrodactyly Syndrome
Epidermolysis Bullosa
Ichthyosis Follicularis
Turner Syndrome;
Disorders of Lid Aperture, Congruity, Dynamics
Blink-Related
Parkinson’s Disease
Ocular Surface-Related
Evaporative Dry Eye
Allergic Eye Disease
Vitamin A Deficiency
Short Breakup Time Dry
Eye
Iatrogenic Disease

### Primary Sjögren’s Syndrome

SS is a relatively common systemic autoimmune rheumatic disease, in which lymphocytic infiltration of salivary and lacrimal glands leads to immune-mediated secretory dysfunction.^17^ The prevalence of PSS in the USA has been estimated to be 0,6-1%, affecting between 0,4 million to 3,1 million adults. SS occurs predominantly in women, with a female-to-male ratio of 9:1. The lacrimal and salivary glands are major targets of the epithelitis, leading to gland destruction and the key symptoms of DED and dry mouth (sicca). The ocular symptoms include blurred vision, grittiness and ocular discomfort and clinical signs include tear film instability, corneal and conjunctival staining, goblet cell loss and epithelial metaplasia.^9^ In addition to sicca syndrome and swollen salivary glands, systemic features, which are included in **Table 5**,^9^ manifest in the majority of patients, and are severe in 15%, particularly affecting the joints, skin, lungs and peripheral nervous system. A recent meta-analysis estimated a pooled relative risk of 13,76 for the development of non-Hodgkin lymphoma, particularly in PSS patients with parotid enlargement, vasculitis, cryoglobulinaemia and Ro and La antibodies.^17^

**Table 5.** Systemic manifestations of PSS.

**Non-specific features**
Musculoskeletal symptoms
Raynaud’s phenomenon
Symptoms of fatigue
**Exocrine Epitheliitis (glandular)**
Lacrimal and Salivary Glands
Other glands - pancreas
**Parenchymal Epitheliitis (extraglandular)**
Bronchial, hepatic, renal - peri-epithelial lymphocytic infiltration
**Endocrine Gland Involvement**
Thyroid, adrenals, ovaries
**Immunocomplex-mediated disease**
Vasculitis - affecting small vessels of the skin, nerves, kidney as a result of B-cell hyperactivity)
**Lymphoproliferative**
B-cell lymphoma

Diagnostic Work-Up: Diagnostic work-up of SS is complicated. There is no single diagnostic test for SS; however, autoantibody testing is crucial, as the presence of autoantibodies has always been considered as one of the criteria for SS diagnosis; in particular, anti-SSA antibodies, which are positive in 80–90% of the cases, and anti-SSB positive in 25–40% of cases. In general, anti-SSA/SSB antibodies have been correlated with younger age at diagnosis, longer disease duration, more severe dysfunction of the exocrine glands, recurrent parotid enlargement, higher intensity of the lymphocytic infiltrates and higher prevalence of extragranular manifestations. ANA and RF-positive patients are frequent and other autoantibodies can be found in SS. The autoantibodies that can be found in SS are summarized in **Table 6**.^18^

**Table 6.** Autoantibodies prevalence.

Anti-Ro/SSA	50–70%
Anti-La/SSB	25–40%
Antinuclear antibodies	85–90%
Rheumatoid factor	36–74%
Anti-mitochondrial antibodies	3–13%
Anti-centromere antibodies	3–27%
Anti-CCP	7–10%

An objective evaluation of eye dryness is obtained with Schirmer’s test, which measures the quantity of tears produced by each eye in 5 minutes. Other tests include a slit lamp examination for assessment of tear break-up (TBUT) and ocular surface staining. Xerostomia can be tested by sialometry, which measures the quantity of saliva produced in 15 minutes. Although, histologic examination remains the gold standard for the diagnosis of SS. Minor salivary gland biopsy should be performed in those subjects who do not have diagnostic DE test or who have negative autoantibodies, with a high clinical suspicion of SS. A total description of the diagnostic criteria is shown in **Table 7**.^17^

**Table 7.** American College of Rheumatology (ACR) 2012 Classification Criteria for SS.

The classification of Sjögren’s syndrome, which applies to individuals with signs/symptoms that may be suggestive of SS, will be met in patients who have at least two of the following three objective features: Positive serum anti-SSA (Ro) and/or anti-SSB (La) OR (positive rheumatoid factor AND ANA ≥ 1:320) Labial salivary gland biopsy exhibiting focal sialadenitis, with a focus score {≥ 1 Focus/4mm ^2^ (square mm)}, as assessed and defined by Daniels 2011 Keratoconjunctivitis sicca with ocular staining score ≥3, as described by Whitcher 2009, assuming that the individual is not currently using daily eye drops for glaucoma and has not had corneal surgery or cosmetic eyelid surgery in the last 5 years.
Prior diagnosis of any of the following conditions would exclude participation in Sjögren’s syndrome studies or therapeutic trials because of overlapping clinical features or interference with criteria tests: History of head and neck radiation treatment Hepatitis C infection Acquired immunodeficiency syndrome Sarcoidosis Amyloidosis Graft versus host disease IgG4-related disease

“Pathogenesis of SS”: SS may result from a range of aberrant immune responses to environmental and viral triggers occurring in genetically susceptible individuals. Hormonal environment is also important, as it is recognized that gender steroids act on the meibomian gland, lacrimal gland, conjunctiva and cornea, primary through nuclear and possibly membrane receptors. Specifically, androgen deficiency is associated with DED, while the precise role of estrogens in the physiology of ocular surface and adnexa is unclear. Furthermore, SS involves a loss of immune tolerance, the presentation of autoantigens and dysregulation of both innate and adaptive immune systems.^9^

First of all, genetic susceptibility plays an important role in the etiology of SS. Identified susceptibility genes include interferon regulatory factor 5 (IRF5), signal transducer and activator of transcription 4 (STAT4) and IL-12A, all participating in IFN signaling; B-lymphocyte kinase (BLK) and chemokine receptor 5 (CXCR5), which are important for B-cell function and antibody production and clearance, and the TNPIP1 gene that is involved in the negative regulation of NF-κΒ pathway.^19^ Genetic regulatory mechanisms are also of emerging interest in the pathogenesis of PSS, with abnormalities observed in DNA methylation and microRNAs.^20^

Furthermore, T-cells play a major role in SS inflammation. Patients with SS have been categorized into distinct groups according to whether the T-cell response is mainly of Th1, Th2 or Th17 type. According to Moutsopoulos, Th1 responses are the most common and Th17 reactivity correlates with greater lesion severity.^21^ SS has been identified mostly as a Th1 – dependent autoimmune disease, with increased concentration of IFN-γ in tears, conjunctiva, lacrimal gland and blood. Recently, Th17 cells, which produce IL-17 and IL-21, and their interaction with Th1 cell, seem to play an important role in pathogenesis of SS. Data from animal models have shown a pro-inflammatory role for IL-17 in sialadenitis, while its specific role in lacrimal gland infiltration is still under debate.^9^

On the other hand, B-cell hyperactivity is recognized as a central element of SS. It is manifested by hypergammaglobulinaemia, cryoglobulinaemia, the production of multiple autoantibodies, directed against a-fodrin, the M3 muscarinic receptor, and the ribonucleoprotein components Ro52 and Ro60 (antiRo/SSA) and La (anti-LA/SSB), and the increased risk of developing B-cell lymphoma.^9^ It is significant to note that circulating levels of B-cell activating factor (BAFF), which is primarily induced by type I and II IFNs, are markedly elevated in PSS.^22^ BAFF is a survival factor for plasmablast that could provide an environment for the production of autoantibodies, and plays a critical role in sustaining or maintaining a germinal center (GC) reaction, as well as in establishing follicular dendritic cell networks. Therefore, we can conclude that BAFF could contribute to pathogenesis of PSS.^17^

Finally, epithelial cells play a crucial role in SS pathogenesis; hence, SS is included in the terminal “autoimmune epithelitis”. A contributor to glandular inflammation is the activation of acinar and ductal epithelial cells to perform immune functions and act as APCs whereby they mediate the recruitment and activation of almost all types of immune cells. The factors which trigger epithelial activation are not known. Activated salivary gland epithelial cells express a range of immunomodulatory molecules implicated in innate and acquired immune responses, they can present autoantigens released from exosomal vesicles or apoptotic bodies. Therefore, they play an important role in initiating and perpetuating the local autoimmune process in the salivary glands in SS.

It is important to know that this knowledge about SS is inferred from the study of labial minor salivary gland biopsies and emphasize that more studies are inquired in lacrimal glands.^9,17^

### Secondary Sjögren’s Syndrome

As it was told earlier, SS is referred as “Primary” in patients who do not have an additional systemic rheumatic disease, and “secondary” when immune-mediated sicca syndrome co-exists in patients with RA, SLE, scleroderma or other autoimmune disease. SS frequently coexists with organ-specific autoimmune diseases such as Graves’ disease and Hashimoto thyroiditis.^17,23^ The symptoms and signs of the systemic condition are usually, but not always, present at the time of dry eye diagnosis.

*Rheumatoid Arthritis (RA):* The most frequent ocular manifestation in patients with RA is DED, with up to 45% of patients having clinical features consistent with DE and 38% of patients being symptomatic.^24^ The DE manifestations usually follow joint involvement and frequently occur in patients with quiescent and well-controlled joint manifestations, including the more severe complications such as corneal melting and sclerokeratitis.^10^ It should be mentioned that, although the ocular manifestations associated with ankylosing spondylitis and psoriatic arthritis are similar, such as anterior uveitis, this differs from RA where DED, peripheral ulcerative keratitis and scleritis are the major ocular manifestations.^24^

*Systemic Lupus Erythematosus (SLE):* Keratoconjunctivitis sicca is commonly seen in SLE patients. In fact, this is the most common ocular manifestation, found in up to one-third of patients. As a consequence of DE, corneal scarring, ulceration and filamentary keratitis can occur as well as decreased visual acuity.^25^ DED occurs in two peaks: the first in patients aged 20–30, and the second in those aged over 50.^10^ It should be mentioned that some of the clinical and immunological similarity between SS and SLE may have a genetic background. A number of SS-associated gene polymorphisms including the MHC-II, STAT-4, IRF5, BLK and TNIP1 genes are shared with SLE.^9^

*Systemic Sclerosis (SSc)*: SSc is a rare, chronic, systemic connective tissue disease of unknown origin characterized by widespread small vessel vasculopathy, immune dysregulation with autoantibody production, and progressive fibrosis. One of the most frequent ocular features of SSc is DED, which has been identified to occur in 37–79% of patients. DED associated with SSc are supported to be the manifestations of systemic complications of scleroderma or adverse effects of the immunosuppressive treatment applied. Ocular signs may occur at any stage of the disease. Although, several studies have shown weak or no correlation between symptoms and signs of DED.^26^

*Dermatomyositis*: Although dermatomyositis and SS share serologic autoantibodies and genetic polymorphisms, population data about the incident of SS in patients with dermatomyositis is unavailable. In a nationwide cohort study in Taiwan, Chia-Chun Tjeng et al., show a higher incident of SS among patients with dermatomyositis. A history of dermatomyositis was significantly associated with subsequent SS, even after adjusting for age, sex and concomitant RA, SLE and SSc.^27^

*Other disorders*: Other associated disorders include polyarteritis nodosa, Wegener’s granulomatosis, PBC and mixed connective tissue disease.^10^

Last but not least, it is worth mentioning IgG4-RD. Although it should be clarified that IgG4-RD is not included in the spectrum of secondary SS, as exclusion of IgG4-RD is necessary in the Classification Criteria for SS. IgG4-RD is a recently described entity and consists a fibro-inflammatory systemic disease characterized by IgG4-positive plasma cell infiltration and obliterative phlebitis. Orbital involvement in IgG4-RD is common, and the orbit was the first extrapancreatic site to be reported in the literature. Common patterns of IgG4-R ophthalmic disease are dacryoadenitis, orbital soft tissue involvement, enlarged orbital nerves, eyelid lesions and extraocular muscle involvement. The lacrimal gland is the most commonly involved in IgG4-ROD, and more than half of the cases show bilateral lacrimal gland involvement. Therefore, DE may potentially develop in patients with orbital disease because of lacrimal gland involvement, proptosis and orbital nerve involvement.^28^

### Clinical features of Dry Eye

The major symptoms of DE are that of dryness, grittiness and burning that worsens during the day. A stinging sensation, transient blurring of vision, redness and crusting of the eyelids are also common; less frequent symptoms include itching, photophobia and a tired heavy feeling of the eyes.^10^ Symptoms are subjective and increase in special environmental conditions, such as wide exposure, dry heat and low humidity, and in the presence of contact lens, smoking, air conditioning or heating.^29^

As we understand, DED affects both vision and comfort of the eye. The source of visual symptoms that occur in the interblink interval is the tear film instability and break up and as well, epithelial roughness in regions of tear break-up. The basis for the symptoms of discomfort is tear hyperosmolarity, while for the symptoms associated with friction and reduced lubrication, is low tear volume, loss of goblet cells and loss of mature glycocalyx, punctate epithelial keratitis and filamentary keratitis. Filamentary keratitis in combination with inflammatory mediators and neurosensory factors such as trigeminal hypersensitivity and neuropathic firing, are the basis for the pain in DED.^9^ All these sources of the DE symptoms are, actually, the signs of DED.

### Diagnostic algorithm

**Figure 1.**
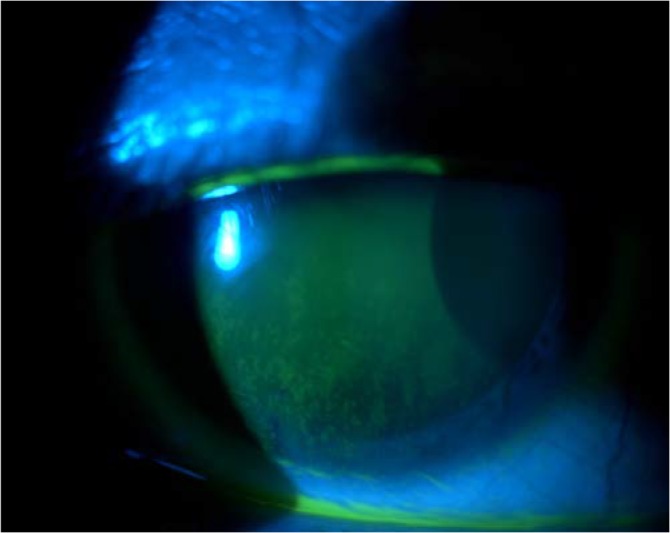
Corneal staining. Superficial Punctate keratitis due to Dry Eye Disease. *Archives of Dr Nikolaos Trakos

**Figure 2.**
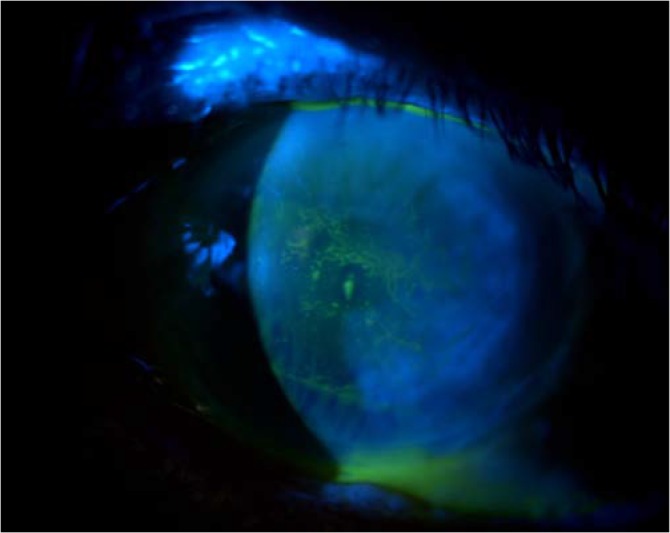
Staining of the ocular surface with fluorescein in Dry Eye Disease. This patient has blurry vision due to Dry Eye.

The clinical diagnosis of DE is based on the presence of classical symptoms and signs. The diagnostic algorithm includes tests to quantity patients’ symptoms, visual disturbance, tear film instability, osmolarity, tear volume, ocular surface damage, inflammation of the ocular surface and eyelid signs (such as MGD).^3^ The diagnostic work-up is summarized below:

1. *Symptoms Questionnaires*: These questionnaires are an excellent opportunity for screening patients with potential dry eye, and a positive symptom score should then trigger a more detailed examination for clinical signs of DED.^3^ The most commonly used in an ophthalmology setting is the ocular surface Disease Index (OSDI), measuring the severity of DED in the form of a 12-item questionnaire subdivided into 3 domains (visual function, ocular symptoms, environmental triggers), providing a scoring algorithm ranging from 100 for complete disability to 0 for no disability.^24^
2. *Ocular surface staining*: Three main dyes are used in the diagnosis of DE. Fluorescein 2% stains corneal and conjunctival epithelial defects and is very accurate in revealing surface damage secondary to tear film defects (**Figures 1, 2**). In contrast, Rose Bengal 1% has a high affinity for dead or devitalized epithelial cells within an altered mucous layer, enabling mucous filaments and plaques on the cornea readily to be stained pink by this dye.^30^ Lissamine green also identifies devitalized epithelial cells present on an intact ocular surface. The ocular staining score (OSS) is an elaborate system gaining acceptance for scoring severity of ocular dryness in DED and uses both lissamine and fluorescein vital dyes. Lissamine is reserved for evaluating the conjunctival staining score and fluorescein for corneal staining.^24^
3. *Tear film stability*: The tear film break-up time (TBUT) measures the stability of the tear film and how quickly this evaporates. It is defined as the interval between the last complete blink and the first appearance of a dry spot or disruption in the tear film. A TBUT ≥10 sec is considered to be normal, and ≤ 5 sec is reduced.^24^
4. *Reflex Tear Flow – The Schirmer Test*: Schirmer’s test I without anaesthetic is the rheumatology “Gold Standard” for quantitave measurement of tear production.^24^ In this test, a paper strip is inserted over the lower lid margin at the junction of its middle and outer thirds. The eye is kept closed and the amount of wetting is measured after 5 minutes. The cut-off for diagnosing DED is wetting of ≤ 5–5,5 mm at 5 minutes. This test can also be performed in the presence of topical anaesthesia, when the cut-off used is usually 10 mm.^31^ Furthermore, Schirmer’s II test is similar, but involves induction and measurement of “reflex” secretion by anaesthetizing the eye and irritating the nasal mucosa.^24^
5. *Hyperosmolarity*: Osmolarity tear analysis is determined by lab-on-a-chip technology using a nanolitre collective pool providing an absolute numerical measurement with a mean average of >308 Osm/L generally indicative of DED. Because of the large overlap between the normal distribution curves between those with and without DE, longitudinal follow-up of patients is essential to monitor changes.^24^

### Therapeutic Approach

The management of DED is complicated, due to its multifactorial etiology. Clinicians must take their best efforts to identify the degree to which EDE or ADDE contribute to the patient’s presentation. It is important to identify and treat the primary source of the disease, while the ultimate aim of DED management is to restore the homeostasis of the ocular surface and tear film, through breaking the vicious circle of DED. So, different approaches can be taken to treat DE depending on the cause and disease severity. These are:

*General measures,* which include reduction of exposure to smoky environments, dust and air conditioning, as these are all situations in which the evaporation rate of tear film is increased. Patients should be advised to take care of their eyelid hygiene and use warm compresses. These last two measures in combination with use of anti-inflammatory therapy may control stagnated meibum oils, hypercolonisation by staphylococci, chronic inflammation, hyperkeratinisation, cicatrisation and the blockage of the Meibomian gland.^24^ There is also evidence that a healthy diet rich in ω-3 fatty acids is helpful.^3^

*Tear film Supplementation* is designed primarily to reduce biomechanical trauma caused by DE and dilute toxic mediators on the ocular surface. These supplements do not replace the intricate composition of the tear film. For mild DED, preserved eye drops may be used, but dosing is critical. If dosing is more than 4–6 drops per day, non-preserved tear supplementation and those not containing BAK should be administered into the eye, because BAK damages the ocular surface epithelium causing ocular surface inflammation and paradoxical aggravation of disease.^32^ The main variables in the formulation of ocular lubricants concern the concentration and choice of electrolytes, osmolarity and the type of viscosity/polymeric system.^10^ In refractive cases, natural biological fluids can be used to substitute for natural tears, including serum and saliva, autologous or allogenic. These biological substitutes contain various epitheliotrophic factors and support corneal epithelium better than pharmaceutical options, but are still worse than normal tears.^3^

*Tear retention* can be achieved by punctal occlusion with either punctal plugs or cautery. Punctal occlusion can increase proinflammatory cytokines expression so timing of occlusion is critical.^33^ It represents a more radical treatment for moderate to severe cases that do not improve with topical lubricants. Other methods of increasing tear retention include the use of moisture chamber spectacles or some specialized types of contact lenses, including silicone rubber lenses and gas-permeable sclera-bearing contact lenses.^34^

*Tear stimulation*: Several agents may increase tear production such as P2Y2 agonist, diquafosol, and oral pilocarpine, a cholinergic agonist, which improves symptoms and possibly increases goblet cell density in the conjunctiva. However, use is frequently limited by intolerable systemic cholinergic symptoms.^24^

*Anti-inflammatory therapy*: Regardless of the initiating cause, a vicious circle of inflammation can develop on the ocular surface in DE that leads to ocular surface disease, and a number of anti-inflammatory agents have been shown to be of benefit in treating this.

Topical corticosteroids target the inflammatory component, but ophthalmological surveillance is mandatory because of the risk of steroid-induced raised intraocular pressure or cataract.^24^ The duration of topical corticosteroids should be limited, unless it concerns refractive cases, in which the duration can be longer.^3^

The anti-MMP properties of long-term oral tetracyclines (such as 50mg once daily for a minimum of 6 weeks) provide anti-inflammatory (anti- TNF, IL-1) treatment. They are, also, antibacterial and antiangiogenic, so they promote an optimal ocular microenvironment in DED.

In 2003, cyclosporine ophthalmic emulsion 0,05% was the first FDA-approved prescription medication for DED, as well as the first to modify disease rather than to act as palliative measure as lubricants do. Cyclosporine is a lipophilic cycle polypeptide, which inhibits T-cell activation and down regulates inflammatory cytokines in the conjunctiva and lacrimal gland, enhancing tear production. There is also evidence that it increases conjunctival goblet cell density and decreases epithelial cell apoptosis. The most common side effect of cyclosporine is ocular burning, while other side effects include blurred vision, ocular itching, conjunctival hyperemia and foreign body sensation.^35^

Lifitegrast, an ICAM inhibitor, has recently been approved by the FDA for the treatment of DED.^9^

In regards to systemic therapy, the treatment of SS with disease-modifying agents (DMARDs) is mainly empirical, and evidence-based recommendations for the treatment are lacking, and no therapy has shown to significantly affect disease course. Hydroxychloroquine (200–400mg/d) is useful for extragranular manifestations, however, recent clinical trials showed that it was no better in improving dryness. The results for the use of methotrexate are similar. Biologics targeting TNF-a have failed to achieve primary outcomes in SS, while rituximab (anti-CD20 B-cell depleting therapy) demonstrates promising results in the treatment of SS. The BAFF antagonist (belimumab) currently approved for SLE, has been evaluating in SS, and recent data suggest that long-term use is effective in reducing disease activity. In conclusion, it is not clear which patients could benefit most of biologic therapy.^18^

## ABBREVIATIONS

ADDE: Aqueous-Deficient Dry Eye
ANA: Antinuclear antibodies
APC: Antigen-presenting cells
BAFF: B-cell Activating Factor
BAK: Benzalkonium chloride
DED: Dry Eye Disease
DMARDs: Disease-modifying antirheumatic drugs
EDE: Evaporative Dry Eye
EMT: Epithelial- Mesenchymal Transition
GVHD: Graft-versus-host disease
HRQoL: Health Related Quality of Life
ICAM: Intracellular Adhesion Molecule
IFN: Interferon
IL: Interleukin
LFA: Integrin Leukocyte Function Antigen
LFU: Lacrimal Functional Unit
lgG4-RD: lgG4-Related Systemic Disease
MAP Kinase: Mitogen-Activated Protein Kinase
MMP: Matrix Metalloproteinases
NF-κβ: Nuclear Factor κB
NK: Natural Killer
PBC: Primary biliary cholangitis
PSS: Primary Sjögren’s Syndrome
RA: Rheumatoid arthritis
RF: Rheumatoid factor
SLE: Systemic Lupus Erythematosous
SS: Sjögren’s Syndrome
SSc: Systemic sclerosis
STAT: Signal transducer and activator of transcription
TBUT: Tear breakup time
Th: T helper
TNF: Tumor Necrosis Factor
